# 438. Phenotypic Differences Between Distinct Immune Biomarker Clusters During the ‘Hyperinflammatory’ Middle-Phase of COVID-19

**DOI:** 10.1093/ofid/ofab466.637

**Published:** 2021-12-04

**Authors:** Paul W Blair, Joost Brandsma, Nusrat J Epsi, Stephanie A Richard, Deborah Striegel, Josh Chenoweth, Rittal Mehta, Emily Clemens, Allison Malloy, Charlotte Lanteri, J Stephen Dumler, David Tribble, Timothy Burgess, Simon Pollett, Brian Agan, Danielle Clark

**Affiliations:** 1 Uniformed Services University, Bethesda, Maryland; 2 Henry M. Jackson Foundation, Bethesda, Maryland; 3 HJF, Bethesda, Maryland; 4 Infectious Disease Clinical Research Program, Department of Preventive Medicine and Biostatistics, Uniformed Services University of the Health Sciences, Bethesda, MD and Henry M. Jackson Foundation, Bethesda, MD, Bethesda, Maryland; 5 Walter Reed National Military Medical Center, Bethesda, Maryland; 6 Infectious Disease Clinical Research Program, Uniformed Services University of the Health Sciences, Boyds, Maryland; 7 Uniformed Services University of the Health Sciences, Bethesda, MD; 8 Infectious Disease Clinical Research Program, Bethesda, Maryland; 9 Infectious Disease Clinical Research Program, USU/HJF, Bethesda, Maryland

## Abstract

**Background:**

Severe acute respiratory syndrome coronavirus 2 (SARS-CoV-2) infections peak during an inflammatory ‘middle’ phase and lead to severe illness predominately among those with certain comorbid noncommunicable diseases (NCDs). We used network machine learning to identify inflammation biomarker patterns associated with COVID-19 among those with NCDs.

**Methods:**

SARS-CoV-2 RT-PCR positive subjects who had specimens available within 15-28 days post-symptom onset were selected from the DoD/USU EPICC COVID-19 cohort study. Plasma levels of 15 inflammation protein biomarkers were measured using a broad dynamic range immunoassay on samples collected from individuals with COVID-19 at 8 military hospitals across the United States. A network machine learning algorithm, topological data analysis (TDA), was performed using results from the ‘hyperinflammatory’ middle phase. Backward selection stepwise logistic regression was used to identify analytes associated with each cluster. NCDs with a significant association (0.05 significance level) across clusters using Fisher’s exact test were further evaluated comparing the NCD frequency in each cluster against all other clusters using a Kruskal-Wallis test. A sensitivity analysis excluding mild disease was also performed.

**Results:**

The analysis population (n=129, 33.3% female, median 41.3 years of age) included 77 ambulatory, 31 inpatient, 16 ICU-level, and 5 fatal cases. TDA identified 5 unique clusters (Figure 1). Stepwise regression with a Bonferroni-corrected cutoff adjusted for severity identified representative analytes for each cluster (Table 1). The frequency of diabetes (p=0.01), obesity (p< 0.001), and chronic pulmonary disease (p< 0.001) differed among clusters. When restricting to hospitalized patients, obesity (8 of 11), chronic pulmonary disease (6 of 11), and diabetes (6 of 11) were more prevalent in cluster C than all other clusters.

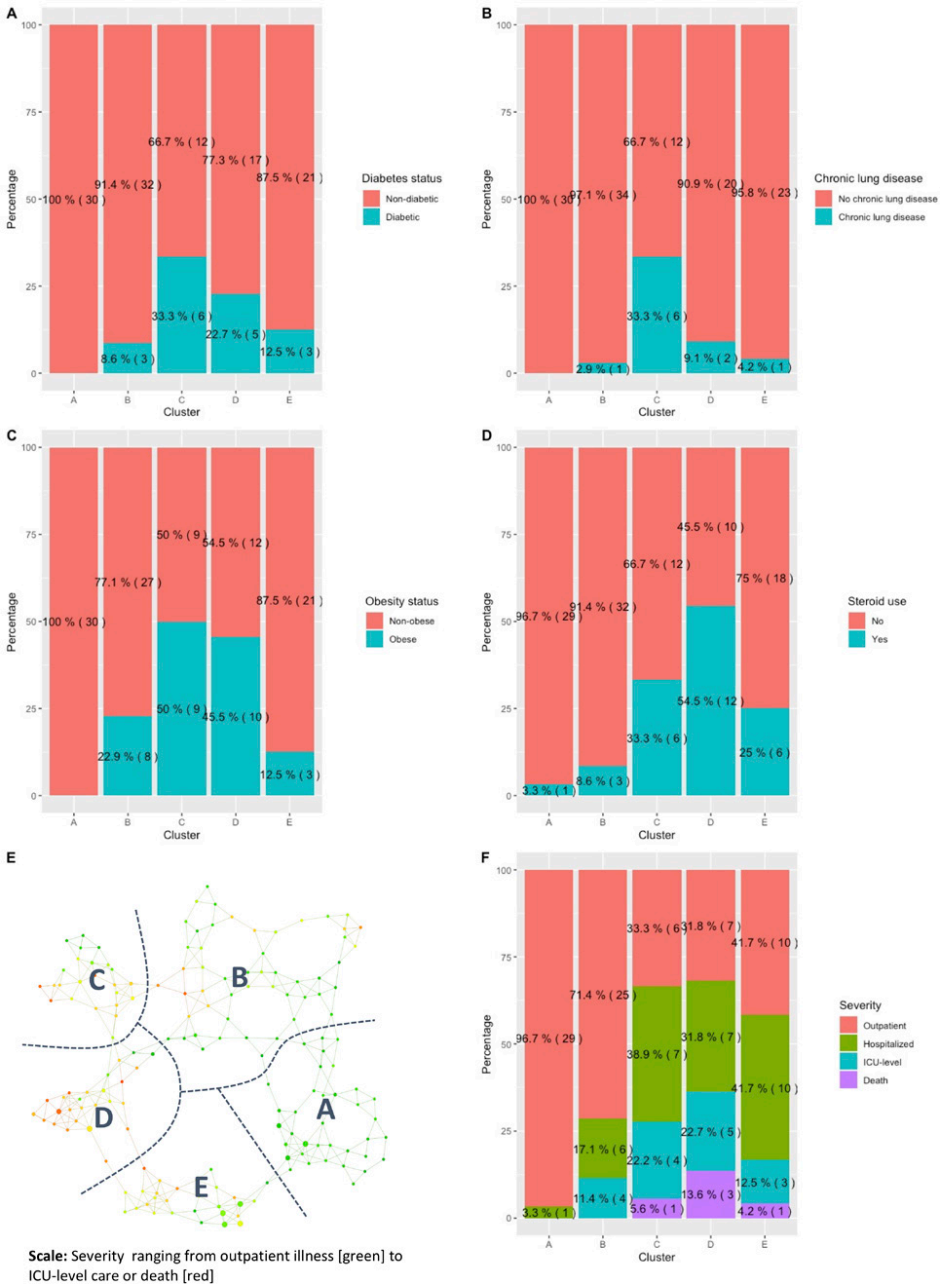

Cluster differences in comorbid diseases and severity by cluster. 1A: bar plot of diabetes prevalence; 1B: bar plot of chronic lung disease ; 1C: bar plot of obesity prevalence; 1D: prevalence of steroid treatment ; 1E: Topologic data analysis network with clusters labeled; 1F: Bar plot of ordinal levels of severity.

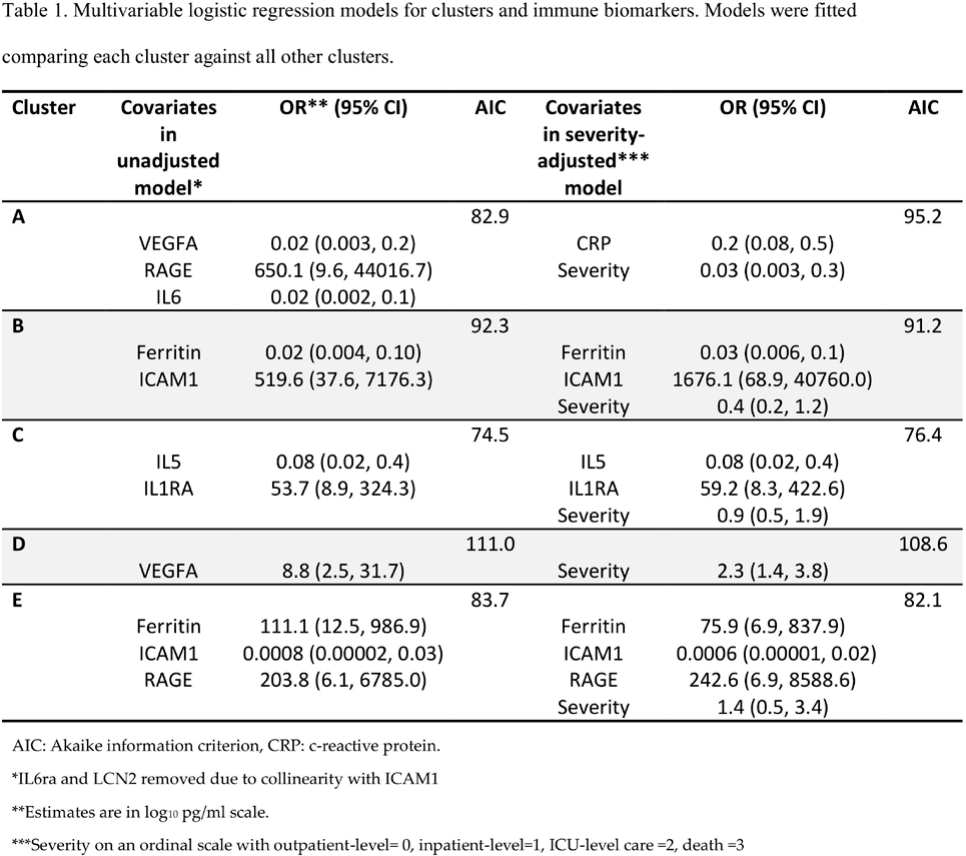

**Conclusion:**

Machine learning clustering methods are promising analytical tools for identifying inflammation marker patterns associated with baseline risk factors and severe illness due to COVID-19. These approaches may offer new insights for COVID19 prognosis, therapy, and prevention.

**Disclosures:**

**Simon Pollett, MBBS**, **Astra Zeneca** (Other Financial or Material Support, HJF, in support of USU IDCRP, funded under a CRADA to augment the conduct of an unrelated Phase III COVID-19 vaccine trial sponsored by AstraZeneca as part of USG response (unrelated work))

